# Content-Based Image Retrieval Using Colour, Gray, Advanced Texture, Shape Features, and Random Forest Classifier with Optimized Particle Swarm Optimization

**DOI:** 10.1155/2022/3211793

**Published:** 2022-04-21

**Authors:** Manoharan Subramanian, Velmurugan Lingamuthu, Chandran Venkatesan, Sasikumar Perumal

**Affiliations:** ^1^Department of Computer Science, School of Informatics and Electrical Engineering, Institute of Technology, Hachalu Hundessa Campus, Ambo University, Ambo, Post Box No.: 19, Ethiopia; ^2^Dr. N.G.P. Institute of Technology, Coimbatore-641407, Tamilnadu, India; ^3^Department of Computer Science, Kombolcha Institute of Technology, Wollo University, Ethiopia

## Abstract

In this paper, a new approach for Content-Based Image Retrieval (CBIR) has been addressed by extracting colour, gray, advanced texture, and shape features for input query images. Contour-based shape feature extraction methods and image moment extraction techniques are used to extract the shape features and shape invariant features. The informative features are selected from extracted features and combined colour, gray, texture, and shape features by using PSO. The target image has been retrieved for the given query image by training the random forest classifier. The proposed colour, gray, advanced texture, shape feature, and random forest classifier with optimized PSO (CGATSFRFOPSO) provide efficient retrieval of images in a large-scale database. The main objective of this research work is to improve the efficiency and effectiveness of the CBIR system by extracting the features like colour, gray, texture, and shape from database images and query images. These extracted features are processed in various levels like removing redundancy by optimal feature selection and fusion by optimal weighted linear combination. The Particle Swarm Optimization algorithm is used for selecting the informative features from gray and colour and texture features. The matching accuracy and the speed of image retrieval are improved by an ensemble of machine learning algorithms for the similarity search.

## 1. Introduction

Over the last few years, the growth of digital images on the World Wide Web has been increased extensively. Users in various specialized fields are exploiting the opportunities provided by the capability to access and control remotely stored images in all categories. In such a situation, it is complex for users to construct effective and improved [1] CBIR (Content-Based Image Retrieval) techniques for the image retrieval process with largely collected images. This research proposes a content-based image retrieval system with the concept of advanced improved filtering and a novel feature extraction process.

Content-Based Image Retrieval (CBIR) has become a significant area of research with the ever-increasing demand and use of digital images in different fields such as medicine, government databases, sciences, and digital photography. The explosive growth of the internet and the wide use of digital content demand the development of effective ways of managing the visual information by its content and have enlarged the necessities for efficient image retrieval procedures. In earlier research, the relevant images in the database for query image are retrieved based on the Mahalanobis distances with the extraction of features [2] such as colour feature, gray feature, colour texture feature, and gray texture feature. However, it is essential to add new features in the future for better retrieval efficiency. To address this, the current research proposes an efficient CBIR method by extracting additional shape feature with the features used in earlier work [3].

The shape is one of the essential visual and primitive features which are used for image content description. Preprocessing of the image is done by implementing high-level filtering techniques such as the Anisotropic Morphological Filters, Kalman Filters, and Particle Filters proceeding with the feature extraction method [4]. Shape features and shape invariant features are computed by using contour-based shape feature extraction methods and image moment extraction methods. The extracted feature has been selected and combined by using Particle Swarm Optimization (PSO). The Random Forest (RF) classifier [5] is used for classifying the database images based on the training images. RF works well when several features are available and provide efficient retrieval of images in large-scale databases. The proposed methods have experimented with the images from both NUS-WIDE and PASCAL-VOC datasets. The effectiveness and efficiency of each proposed technique are evaluated by measuring accuracy, precision, recall, and time. These experiments are carried out by using the MATLAB R2015a.

The proposed model within 105 ms achieved an accuracy of 94.67%, a precision of 94.79%, and recall of 94.67% on the 400 test images for the NUS-WIDE dataset. The proposed model within 95 ms achieved an accuracy of 92%, a precision of 92.12%, and a recall of 92% on the 400 test images for the PASCAL-VOC dataset. The results obtained on diverse datasets prove the superiority and robustness of the proposed work. The rest of the paper is organized as follows: Section 2 illustrates this research's related and existing works, and Section 3 describes the proposed methodology.  Section 4 gives the details of the results and discussion. Then, Section 5 concludes this research work and gives the direction for the proposed work.

## 2. Related Work

The CBIR concept is used in a lot of fields like biomedicine fields such as X-ray, CT, medical diagnosis, government and security filtering, art galleries, museums, and personal albums. Several previous works have been done for solving various feature extraction methods of the image elements for image retrieval. This section deals with some related and existing works that have been done in this research area. Awang et al. [2] proposed feature extraction techniques using CT brain image (grayscale image). Kumar and Esther [4] analyzed a trial of employing it in colour images with various feature extraction methods like colour histogram (colour feature), Gabor transform (texture feature), and Wavelet transform (texture feature) and also with distance metric measures like Euclidean distance, Chi-square distance, and weighted Euclidean distance. A. Rashno and E. Rashno [1] developed a new feature extraction schema including the norm of low-frequency components in wavelet transformation and colour features in RGB and HSV domains which are proposed as a representative feature vector for images in database followed by appropriate similarity measure for each feature type. Thilagam and Arunesh [6] presented a framework that permits classifying medical images to recognize conceivable diseases that are affected. This is done by image retrieval from the collection of the dataset by inputting the query image.

Latif et al. [7] aimed to present a comprehensive review of the recent development in the area of CBIR and image representation. We analyzed the main aspects of various image retrieval and image representation models from low-level feature extraction to recent semantic deep-learning approaches. The important concepts and major research studies based on CBIR and image representation are discussed in detail, and future research directions are concluded to inspire further research in this area. Suresh et al. [8] described a hybrid feature extraction approach of the research and solution to the problem of designing a CBIR system manually. Two features are used for retrieving the images such as colour and texture. The colour feature is extracted by using different colour spaces such as RGB, HSV, and YCbCr. The texture feature is extracted by applying gray-level cooccurrence matrix (GLCM). The image is retrieved by combining colour and texture features and the colour space which gives the best result as analyzed using precision and recall graph. In this research work, a new improved CBIR system has been addressed by extracting colour, gray, advanced texture, and shape features for input query images. Contour-based shape feature extraction methods and image moment extraction techniques are used to extract the shape features and shape invariant features. The informative features are selected from extracted features and combined colour, gray, and texture and shape features by using PSO. The proposed method of CBIR gives better results in terms of accuracy, precision, recall, and execution time than the existing methods.

Hameed et al. [9] analyzed and compared the current state-of-the-art methodologies over the last six years in the CBIR field. This paper also provided an overview of the CBIR framework, recent low-level feature extraction methods, machine learning algorithms, similarity measures, and a performance evaluation to inspire further research efforts. Latif et al. [10] aimed to present a comprehensive review of the recent development in the area of CBIR and image representation. Further, they analyzed the main aspects of various image retrieval and image representation models from low-level feature extraction to recent semantic deep-learning approaches. The important concepts and major research studies based on CBIR and image representation are discussed in detail, and future research directions are concluded to inspire further research in this area. Abdullah et al. [11] discussed and described the colour features technique for image retrieval systems. Several colour features technique and algorithms produced by the previous researcher are used to calculate the similarity between extracted features. This paper also described the specific technique about the colour basis features and combined features (hybrid techniques) between colour and shape features.

Singh et al. [12] developed a fast and effective CBIR system that uses supervised learning-based image management and retrieval techniques. It utilizes machine learning approaches as a prior step for speeding up image retrieval in the large database. For the implementation of this, first, we extract statistical moments and the orthogonal combination of local binary pattern- (OC-LBP-) based computationally light-weighted colour and texture features. Further, using some ground truth annotation of images, we have trained the multiclass support vector machine (SVM) classifier. This classifier works as a manager and categorizes the remaining images into different libraries. However, at the query time, the same features are extracted and fed to the SVM classifier. SVM detects the class of query and searching is narrowed down to the corresponding library. This supervised model with the weighted Euclidean Distance (ED) filters out maximum irrelevant images and speeds up the searching time. This work is evaluated and compared with the conventional model of the CBIR system on two benchmark databases, and it is found that the proposed work is significantly encouraging in terms of retrieval accuracy and response time for the same set of used features. Nasim and Shervan [13] described a new approach for content-based image retrieval based on a weighted combination of colour and texture features. Firstly, to achieve discriminant features, texture features are extracted using modified local binary patterns (MLBP) and local neighborhood differences patterns (LNDP) and filtered gray-level cooccurrence matrix (GLCM). Also, a quantization colour histogram is used to extract colour features. Next, similarity matching is performed based on the Canberra distance in colour and texture features separately. Finally, a weighted decision is performed to retrieve the most similar database images to the user query. Nazgol and Fekri-Ershad [13] presented a method for image retrieval based on a combination of local texture information derived from two different texture descriptors. First, the colour channels of the input image are separated. The texture information is extracted using two descriptors such as evaluated local binary patterns and predefined pattern units. After extracting the features, similarity matching is done based on distance criteria. The performance of the proposed method is evaluated in terms of precision and recall on the Simplicity database.

## 3. Proposed Methodology

**Figure 1:**
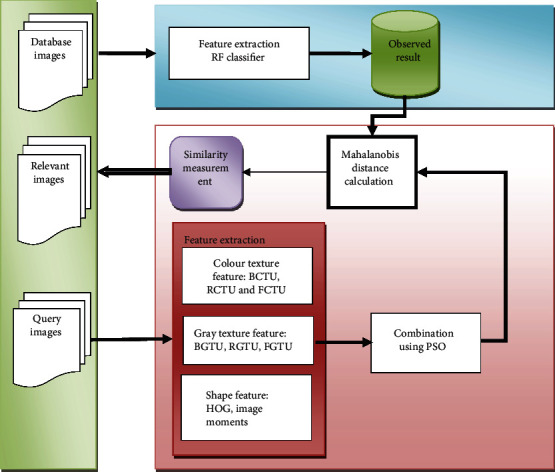
The framework of proposed CGATSFRFOPSO method.

Figure 1 illustrates the framework of proposed colour, gray, advanced texture, shape feature, and random forest classifier with optimized PSO (CGATSFRFOPSO) approach. The following section furnishes the overview of proposed shape features and their extraction procedure.

### 3.1. Texture-Based Feature Extraction

The texture is a significant spatial feature that is useful for identifying regions of interest in an image. Various texture-based methods are developed for extracting features of the images. For an effective image retrieval system, especially with poor illumination of images, resolution levels and noises, the advanced texture-based feature extraction technique is used for the selection of appropriate and efficient similarity features. The current work deals with extracting colour texture features, gray-level texture features, and texture units. These extracted texture units of colour and gray-level texture features are Basic Texture Unit (BTU), Reduced Texture Unit (RTU), and Fuzzy-Based Texture Unit (FTU). These units give out the textural information with complete texture characteristics in all directions instead of working with only one displacement vector. The texture feature consists of two components, namely, the gray texture feature and the colour texture feature. The proposed method integrates these two features by using a cooccurrence matrix and feature extraction with optimized PSO.

#### 3.1.1. Gray Texture Feature Extraction

The gray texture feature can be extracted by using gray-level cooccurrence matrix which estimates gray-level relationships between the pixels of the image. In statistical image analysis, gray-level cooccurrence matrix (GLCM) is a common technique used to estimate image properties that are related to second-order statistics. In one offset, the relation between two neighboring pixels is considered the second-order texture in which the first pixel is called as a reference and the second one as the neighbor pixel. GLCM is said to be a joint probability of two-dimensional matrix *P*_d,*θ*_(*i*, *j*) among the pair of pixels which splits by the distance (*d*) in a specified direction *θ*. By using GLCM, gray texture feature can be extracted by using Homogeneity for feature vector estimation. Homogeneity can be defined as follows:

(1) $$\begin{array}{c} {\text{Homogeneity}}_{\text{d},\theta }=\underset{i,j}{\sum }\frac{P_{d,\theta }\left(i,j\right)}{1+\left|i-j\right|}. \end{array}$$

The texture unit can be illustrated by taking the relative gray-level relationships between central pixel and its neighboring pixels. Gray-level texture can be decomposed into a set of Gray Texture Units (GTU). These texture units represent statistical texture units and local texture aspect in an image for revealing gray-level texture information. The three different texture units for the gray-level image can be represented as Basic Gray Texture Unit (BGTU), Reduced Gray Texture Unit (RGTU), and Fuzzy Gray-Based Texture Unit (FGTU).

The three texture units for gray-level texture feature can be represented as follows:

(2) $$\begin{array}{c} N_{\text{BGTU}}=\overset{8}{\underset{i=1}{\sum }}E_{i}\times 3^{i-1},N_{\text{BGTU}}\in \left\{0,1\cdots .\left(3^{8}-1\right)\right\}, \end{array}$$

where *N*_BGTU_ denotes the number of basic gray-level texture units.

(3) $$\begin{array}{c} N_{\text{BGTU}}=\overset{8}{\underset{i=1}{\sum }}E_{i}\times 2^{i-1},N_{\text{RGTU}}\in \left\{0,1\cdots \left(2^{8}-1\right)\right\}, \end{array}$$

where *N*_RGTU_ denotes the number of reduced gray-level texture units and

(4) $$\begin{array}{c} N_{\text{FGTU}}=\overset{8}{\underset{i=1}{\sum }}E_{i}\times 5^{\left(\frac{\text{i}-1}{2}\right)}, \end{array}$$

where *N*_FGTU_ denotes the number of Fuzzy ray-level texture units, respectively.

#### 3.1.2. Colour Texture Feature Extraction

Colour images can be represented by HSV and RGB colour space. Colour texture feature extraction is done by two types of RGB representations. The first one computes feature vector (FV) from the extracted feature of the RGB channels.

(5) $$\begin{array}{c} \text{Feature }{\text{vector}}_{1}=\left\{\text{FE}\left(\text{R}\right),\text{FE}\left(\text{G}\right),\text{FE}\left(\text{B}\right)\right\}. \end{array}$$

The second type estimates the feature vector from the relation between all six combinations of RGB.

(6) $$\begin{array}{c} \text{Feature }{\text{vector}}_{2}=\left\{\text{FE}\left(\text{RGB}\right),\text{FE}\left(\text{RBG}\right),\text{FE}\left(\text{GBR}\right),\text{FE}\left(\text{GRB}\right),\text{FE}\left(\text{BGR}\right),\text{FE}\left(\text{BRG}\right)\right\}, \end{array}$$

where these combinations of colour are computed from

(7) $$\begin{array}{c} \text{RGB}=\text{round }\left({\text{c}}_{1}\text{R}+{\text{c}}_{2}\text{G}+{\text{c}}_{3}\text{B}\right), \end{array}$$

(8) $$\begin{array}{c} \text{RBG}=\text{round }\left({\text{c}}_{1}\text{R}+{\text{c}}_{2}\text{B}+{\text{c}}_{3}\text{G}\right), \end{array}$$

(9) $$\begin{array}{c} \text{GBR}=\text{round }\left({\text{c}}_{1}\text{G}+{\text{c}}_{2}\text{B}+{\text{c}}_{3}\text{R}\right), \end{array}$$

(10) $$\begin{array}{c} \text{GRB}=\text{round }\left({\text{c}}_{1}\text{G}+{\text{c}}_{2}\text{R}+{\text{c}}_{3}\text{B}\right), \end{array}$$

(11) $$\begin{array}{c} \text{BGR}=\text{round }\left({\text{c}}_{1}\text{B}+{\text{c}}_{2}\text{G}+{\text{c}}_{3}\text{R}\right), \end{array}$$

(12) $$\begin{array}{c} \text{BRG}=\text{round }\left({\text{c}}_{1}\text{B}+{\text{c}}_{2}\text{R}+{\text{c}}_{3}\text{G}\right), \end{array}$$

where *c*_1_: *c*_2_: *c*_3_ represents the ratio.

Colour texture can be extracted by using the method called Colour Level Cooccurrence Matrix (CLCM). In this scenario, feature vector is estimated directly from 3D RGB colour space. For distance *d* = 1, the cube of 3 × 3 × 3 size is created. Three CLCM matrices are estimated for every channel. For example, CLCM estimation of channel green is as follows.

(13) $$\begin{array}{c} {\text{CLCMi}}_{\text{i},\text{j}}\left(G\right)=\overset{1}{\underset{m=-1}{\sum }}\overset{1}{\underset{n=-1}{\sum }}\text{r}\text{elation}\left(\text{img}\left(i,j,2\right)\left|\text{img}\right.\left(i+m,j+n,2\right)\right), \end{array}$$

where m∧n ≠0.

(14) $$\begin{array}{c} {\text{CLCMi}}_{\text{i},\text{j}}\left(\text{GR}\right)=\overset{1}{\underset{m=-1}{\sum }}\overset{1}{\underset{n=-1}{\sum }}\text{r}\text{elation}\left(\text{img}\left(i,j,2\right)\left|\text{img}\right.\left(i+m,j+n,1\right)\right), \end{array}$$

(15) $$\begin{array}{c} {\text{CLCMi}}_{\text{i},\text{j}}\left(\text{GB}\right)=\overset{1}{\underset{m=-1}{\sum }}\overset{1}{\underset{n=-1}{\sum }}\text{r}\text{elation}\left(\text{img}\left(i,j,2\right)\left|\text{img}\right.\left(i+m,j+n,3\right)\right), \end{array}$$

where img is an image represented by RGB (Red, Green, Blue) colour space.

Final CLCM feature vector is expressed as follows:

(16) $$\begin{array}{c} \text{Feature Vector}=\left\{\text{FE}\left(\text{R}\right),\text{FE}\left(\text{RG}\right),\text{FE}\left(\text{RB}\right),\text{FE}\left(\text{G}\right),\text{FE}\left(\text{GB}\right),\text{FE}\left(\text{GR}\right),\text{FE}\left(\text{B}\right),\text{FE}\left(\text{BG}\right),\text{FE}\left(\text{BR}\right)\right\}. \end{array}$$

The three different texture units for colour image can be represented as Basic Colour Texture Unit (BCTU), Reduced Colour Texture Unit (RCTU), and Fuzzy Colour Texture Unit (FCTU).

The three texture units for the colour texture feature can be represented as follows:

(17) $$\begin{array}{c} {\text{N}}_{\text{BCTU}}=\overset{8}{\underset{i=1}{\sum }}E_{i}\times 3^{i-1},N_{\text{BCTU}}\in \left\{0,1\cdots .\left(3^{8}-1\right)\right\}, \end{array}$$

where *N*_BCTU_ denotes the number of Basic Colour texture Units.

(18) $$\begin{array}{c} N_{\text{RCTU}}=\overset{8}{\underset{i=1}{\sum }}E_{i}\times 2^{i-1},N_{\text{RCTU}}\in \left\{0,1\cdots \left(2^{8}-1\right)\right\}, \end{array}$$

where *N*_RCTU_ denotes the number of Reduced Colour level Texture Units, and

(19) $$\begin{array}{c} N_{\text{FCTU}}=\overset{8}{\underset{i=1}{\sum }}E_{i}\times 5^{\left(\frac{\text{i}-1}{2}\right)}, \end{array}$$

where *N*_FCTU_ denotes the number of Fuzzy Colour level Texture Units, respectively.

### 3.2. Shape Feature Extraction

In general, characterizing the shape of an object is quite difficult. The shape is frequently characterized in terms of elongated, rounded, etc., in images. The shape is an important and primitive visual feature for describing image content. It contains all the geometrical information of an object in the image which does not change generally, but it will change when the orientation or location of the objects is changed [6]. General shape features are the perimeter, area, eccentricity, symmetry, etc. Very difficult shapes require computer-based processing, whereas still a lot of practical shape description techniques exist to illustrate the shape. The different shape descriptors such as Histogram of Oriented Gradient [14] (HOG) and image moments, namely, moment invariant and Zernike Moments are described in detail. Histogram of Oriented Gradient (HOG) is defined as significant feature descriptors which are used in computer vision and image processing for identifying the objects in images [15].

**Figure 2:**
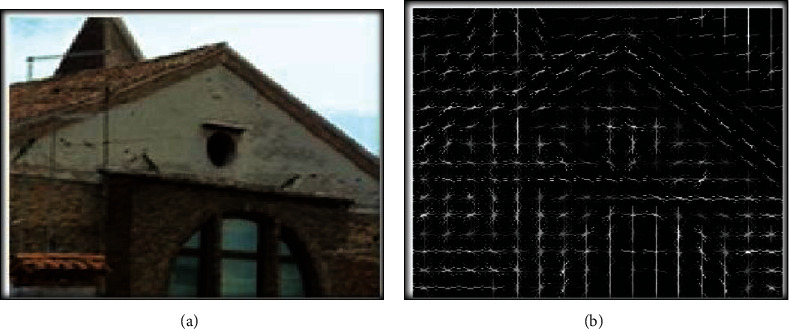
(a) Visual display of an image and (b) visual display of HOG in each cell.

HOG estimates the amount of gradient orientation in localized parts of an image. The basic idea behind the HOG descriptors is that the appearance and shape of the object in an image can be illustrated by the distribution of intensity gradients [16]. The execution of these descriptors can be achieved by separating the image into minute-associated regions known as cells. Each cell interprets the gradient and orientation histograms for the pixels in the cells. The grouping of these histograms characterizes the descriptor.  Figure 2 shows the patch of images with their resultant HOGs [9].

Initially, the gradients and orientations are computed at each pixel in the local region of an image. Gradients and orientations of edge are obtained by using the Sobel Filters. The gradient magnitude and orientation are denoted as *M* (*x*, *y*) and *θ* (*x*, *y*), respectively, which are estimated using the *x* and *y* directional gradients defined as dx (*x*, *y*) and dy (*x*, *y*) calculated by the Sobel filter as follows [17, 18]:

(20) $$\begin{array}{c} \text{M}\left(\text{x},\text{y}\right)=\sqrt{\text{dx}{\left(\text{x},\text{y}\right)}^{2}+\text{ dy}{\left(\text{x},\text{y}\right)}^{2}}, \end{array}$$

(21) $$\begin{array}{c} \theta \left(\text{x},\text{y}\right)=\left\{\begin{array}{c} \tan^{-1}\left(\frac{\text{dy}\left(\text{x},\text{y}\right)}{\text{dx}\left(\text{x},\text{y}\right)}\right)-\pi \text{ if dx}\left(\text{x},\text{y}\right)<0\;\text{and }dy\left(x,y\right)<0\\ \tan^{-1}\left(\frac{dx\left(x,y\right)}{dx\left(x,y\right)}\right)+\pi \text{ if dx}\left(\text{x},\text{y}\right)<0\;\text{and }dy\left(\text{x},\text{y}\right)>0 \end{array}\right\}. \end{array}$$

#### 3.2.1. Image Moments

Various image processing applications such as pattern recognition, image classification, and shape analysis use the image moments. Image Moments is treated as region-based shape descriptors. Image moments such as moment invariant and Zernike Moments (ZM) are explained below.

*(1) Moment Invariants*. Moment invariant is generally used in the application of two-dimensional pattern recognition approaches.

For a digital image *I* (*x*, *y*), the order of two-dimensional moments (*p* + *q*) is defined as follows.

(22) $$\begin{array}{c} \;{\text{m}}_{\text{pq}}=\underset{x}{\sum }\underset{y}{\sum }x^{p}y^{q}I\left(x\left|y\right.\right), \end{array}$$

where *p*, *q* = 0, 1, 2 ⋯ .

The entire image is spanned by using the values of spatial coordinates *x* and *y*. The moment expressed in equation (23) is not invariant under geometrical operations such as scale changes, rotation, or translation in Image I (*x*, *y*). Invariance in translation is obtained by utilizing the central moment, which is defined as follows [3, 19]:

(23) $$\begin{array}{c} \mu_{\text{pq}}=\sum \sum {\left(x-\overset{¯}{x}\right)}^{p}{\left(y-\overset{¯}{y}\right)}^{q}I\;\left(x,y\right) \end{array}$$

where

(24) $$\begin{array}{c} \overset{¯}{x}=m_{10}/m_{00}\;\text{and }\overset{¯}{y}m_{01}/m_{00}. \end{array}$$

The order of *p* + *q* for the normalized central moment is defined as follows:

(25) $$\begin{array}{c} \;\eta_{\text{pq}}=\frac{\mu_{pq}\;}{\mu_{pq}^{Y}}, \end{array}$$

where

(26) $$\begin{array}{c} Y=p+q/2+1. \end{array}$$

*(2) Zernike Moments*. Zernike Moments (ZM) are defined as orthogonal moments which are used to characterize the shape content of an image with a reduced redundancy level of information. These moments permit exact reconstruction of the image and construct best exploitation of image shape information. Zernike Moments (ZM) are extensively used in content-based image retrieval systems as shape descriptors. These Moments have numerous desirable properties such as robust to noise and rotation invariance. The composite ZM is obtained by assigning the image function against an orthogonal polynomial above the interior of a unit circle *x*2 + *y*2 = 1 as follows:

(27) $$\begin{array}{c} V\text{ nm}\left(x,y\right)=V_{nm\left(p,\Theta \right)}=R_{nm\left(\rho \right)\exp\left(\text{jm}\Theta \right)}, \end{array}$$

(28) $$\begin{array}{c} {\text{R}}_{\text{nm}}\left(\rho \right)=\overset{\frac{\left(n-\left|m\right|\right)}{2}}{\underset{x=0}{\sum }}{\left(-1\right)}^{*}\frac{\left(n-s\right)!}{s!\left(-s\right)!\left(n-\left|m\right|/2-s\right)!}\rho^{\text{n}-2\text{x}}, \end{array}$$

where *n* is a nonnegative integer, *m* is an integer so that *n* − *m* is even and *m* < *n*,

(29) $$\begin{array}{c} \rho =\sqrt{x^{2}+y^{2}},\Theta =\tan^{-1}\left(\frac{x}{y}\right). \end{array}$$

Projecting the image methods against the basis set, grades Zernike moments of order *n* with repetition *m* given by.

(30) $$\begin{array}{c} A_{nm}=\frac{n+1}{\pi }\underset{x}{\sum }\underset{y}{\sum }f\left(x,y\right)V_{nm}\left(p,\Theta \right), \end{array}$$

where *x*^2^ + *y*^2^ ≤ 1.

In the proposed work, the shape feature extraction is done by using contour-based shape feature extraction. It is essential to clarify its basis, increase its speed, and enlarge its accuracy and robustness in computer vision. The contour-based shape feature extraction approximates the parameters that govern a shape's appearance, where the shapes range from lines to ellipses and even to unknown shapes.

Contour extraction is a crucial step in the proposed method since shape information is estimated from the contour. An approach of the adaptive thresholding-based method is chosen for estimating the contours of the image. Adaptive thresholding-based methods give sufficient details to describe the overall shape of the image. Adaptive thresholding in the edge-based method is replaced with global thresholding. This may produce more broken boundaries. However, it decreases noisy boundaries successfully, which is the first step towards reaching a better precision rate.

The shape descriptor, namely, Histogram of Oriented Gradient is extracted by using various methods. These features are extracted from the contours of the images. The shape of the contour can be extracted using the following process:

- Square grid representation of image
- Contour-based weighting method

#### 3.2.2. Square Grid Representation of Image

Consider the image of *I*, in which it is represented as a directional code of features. In image *I*, the contour can be extracted using a 4-directional code. From this, directional code for subimages can be obtained by dividing the contour image by a 7 × 7 square grid. Proceeding with this, a histogram of each directional code is attained from each subimage. For each subimage, the obtained histograms are normalized by the total amount of directional codes from the subimages, and then, all histograms are combined and form a feature vector. The Histogram normalization is performed as follows:

(31) $$\begin{array}{c} N_{\text{I}\left(\text{i}\right)}=\frac{N_{\text{I}\left(\text{i}\right)}}{N_{\text{I}\left(\max\right)}}, \end{array}$$

where ${\begin{array}{c} \;\\ \;N \end{array}}_{\text{I}\left(\text{i}\right)}$ is the i^th^ element in a feature vector of *I*^th^ image in a dataset and *N*_*I*(max)_ is obtained by

(32) $$\begin{array}{c} N_{\text{I}\left(\max\right)}=\underset{\text{I}}{\max}{\text{N}}_{\text{I}\left(\text{i}\right)}. \end{array}$$

Different square grids of 3 × 3 to 10 × 10 are tried. Later optimal in terms of retrieval accuracy and dimensionality of the feature vector is found by using 7 × 7. The square grids of 3 × 3 might generally miss the shapes of objects as a relatively large region of objects is covered by one cell. The grid of 10 × 10 does not show better performance than 7 × 7.

#### 3.2.3. Contour-Based Weighting Method

At a time, one contour is considered for the feature extraction task. The images in this research usually produce several tens or hundreds of contours. The number of extracting contours depends on the extraction method. The theory is that a longer contour has more information for shape representation and the longer contour provides an accurate shape of the image. The weight of the image is obtained by concatenating the directional code features with each extracted contour by a distance ratio between its contour length and largest contour length. The longest contour length gives the fine shapes of an image. With this weighting scheme of features, the fine extraction of shape from contour is achieved.

### 3.3. Feature Selection and Optimization Using PSO

Feature selection and combination of these selected features can be done by a well-known optimization technique called Particle Swarm Optimization (PSO). The PSO was developed by Eberhart and Kennedy in which it is an expansion of a social behavior of simulated system that integrates concepts such as nearest-neighbor velocity matching and acceleration by distance.

The current work extends PSO for the combination of different extracted features using colour texture feature and gray texture feature. These extracted features include colour texture feature with the texture units such Basic Colour Texture Unit (BCTU), Reduced Colour Texture Unit (RCTU), and Fuzzy-Based Colour Texture Unit (FCTU) and gray texture feature with the texture unit such as Basic Gray Texture Unit (BGTU), Reduced Gray Texture Unit (RGTU), and Fuzzy-Based Gray Texture Unit (FGTU).

### 3.4. Image Retrieval Using Random Forest Classification

A random forest classifier is an ensemble classifier that consists of several decision trees. The output of this classifier is the class number that most often occurs individually at the output of decision tree classifiers. The main idea of decision trees is to predicate a target based on a group of input data. The decision trees are also named classification trees, where the tree leaves represent the class labels and the branches represent the conjunction of feature vectors that lead to class labels. Each interior node represents an input feature and each node has children of another input feature. The training of the decision tree is based on a process called recursive partitioning and by using this recursive process the input dataset is split into subsets. The recursion ends the condition when all the tree nodes have the same output targets.

The classification-based relevance feedback approach suffers from the problem of imbalanced training dataset, which causes instability and degradation in the retrieval results. In order to tackle with this problem, a novel active learning approach based on random forest classifier and feature reweighting technique is proposed in this paper. Initially, a random forest classifier is used to learn the user's retrieval intention. Then, in active learning, the most informative classified samples are selected for manual labelling and added in training dataset, for retraining the classifier. Also, a feature reweighting technique based on Hebbian learning is embedded in the retrieval loop to find the weights of most perceptive features used for image representation. These techniques are combined together to form a hypothesized solution for the image retrieval problem. The experimental evaluation of the proposed system is carried out on two different databases and shows a noteworthy enhancement in retrieval results.

## 4. Experiment and Result

The performance of the proposed CGATSFRFOPSO method is compared to the CGATMDOPSO method. The parameters taken up for performance comparisons are accuracy, precision, recall, and execution time. The experimented values of the proposed model are tabulated in the tables and the performance is compared with the previous method.

The parameters are calculated for different sizes of database images which are given in Tables 1 and 2, respectively. It is observed from the following tables that the proposed approach provides a better result than the existing method in terms of accuracy, precision, recall, and execution time.

### 4.1. Accuracy

**Figure 3:**
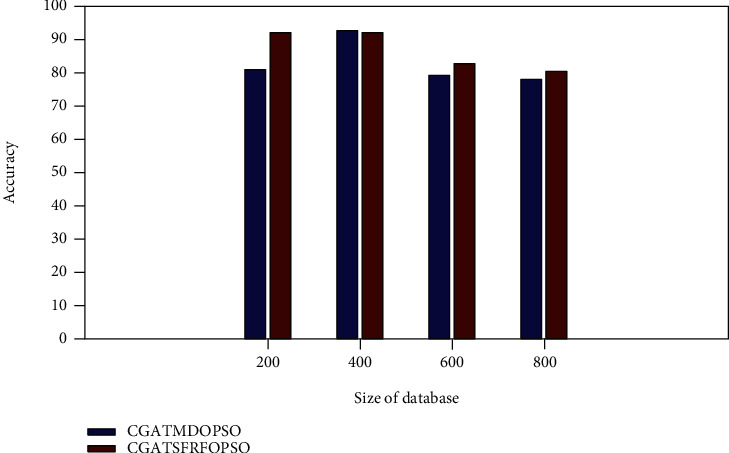
Accuracy comparison (NUS-WIDE).

Figure 3 shows the accuracy comparison of the proposed method with the CGATMDOPSO method. The proposed approach gives the accuracy of 83.7079, 94.6667, 84.4681, and 82.7103 for the NUS-WIDE database size of 200, 400, 600, and 800, respectively. It is observed from Figure 3 that the proposed CGATSFRFOPSO approach provides a better accuracy value than the existing CGATMDOPSO method.

**Figure 4:**
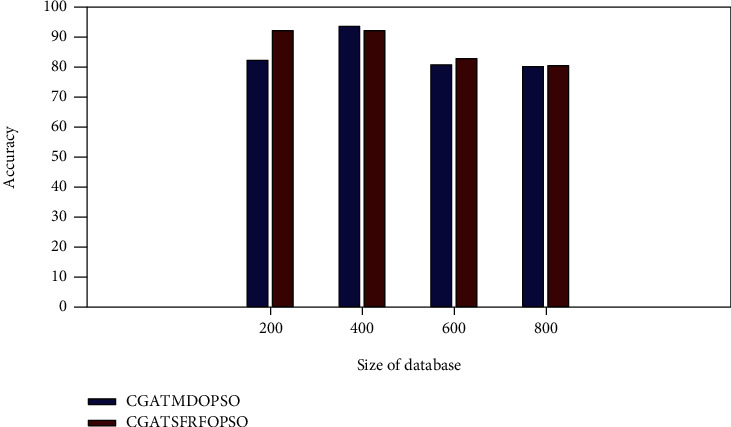
Accuracy comparison (PASCAL VOC).

The accuracy comparison of the proposed method with the CGATMDOPSO method is shown in Figure 4. The proposed CGATSFRFOPSO provides the accuracy value of 82.5843, 92.0000, 82.5532, and 80.3738 for PASCAL VOC database sizes of 200, 400, 600, and 800, respectively. It is a higher accuracy value when compared to the accuracy values obtained by the other two proposed methods.

### 4.2. Precision Rate

**Figure 5:**
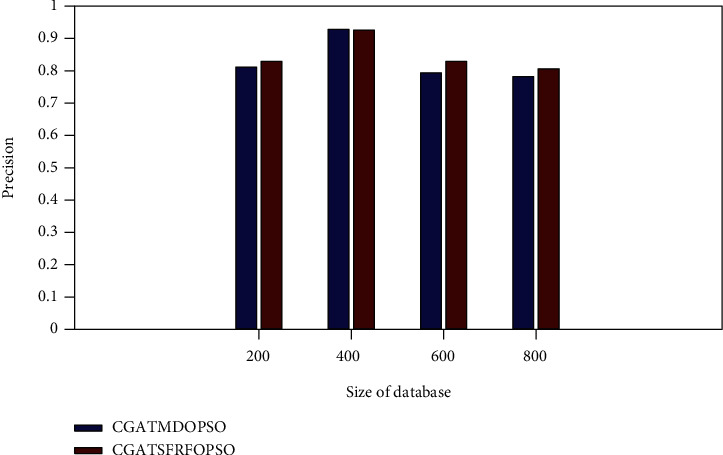
Precision rate comparison (NUS-WIDE).

Figure 5 illustrates that the proposed CGATSFRFOPSO method attains the precision rate of 0.8371, 0.9479, 0.8455, and 0.8263 for the NUS-WIDE database sizes of 200, 400, 600, and 800, respectively. It is higher than the existing CGATMDOPSO method which has the precision rate of 0.8092, 0.9267, 0.7915, and 0.7804, respectively. Thus, the above result confirms that the proposed approach produces a better result than the existing methods.

**Figure 6:**
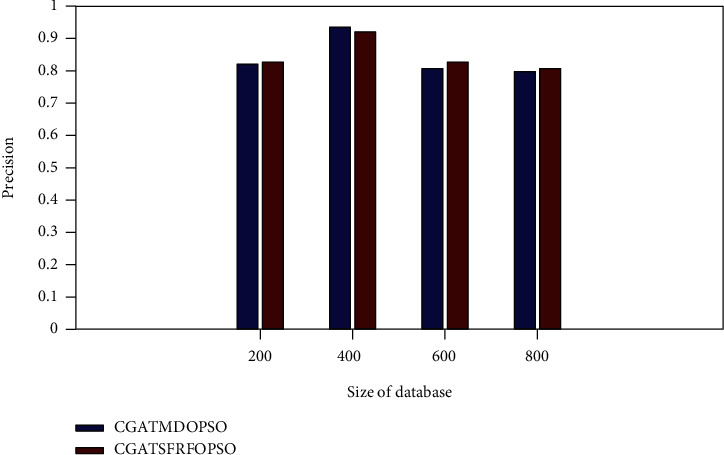
Precision rate comparison (PASCAL VOC).

Figure 6 shows the precision comparison of the proposed method with the existing CGATMDOPSO method. It is observed from Figure 6 that the proposed system obtains a higher precision rate of 0.8258, 0.9212, 0.8258, and 0.8029 for the PASCAL VOC database sizes of 200, 400, 600, and 800, respectively.

### 4.3. Recall Rate

**Figure 7:**
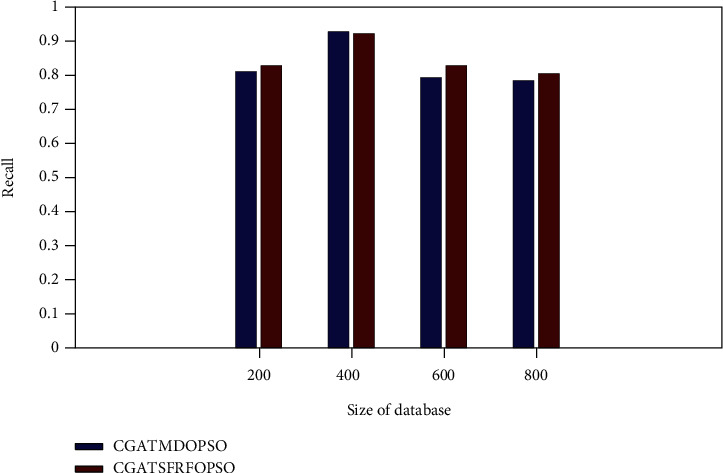
Recall rate comparison (NUS-WIDE).

Figure 7 demonstrates the recall comparison of the proposed method with the CGATMDOPSO method. The proposed system gives the recall value of 0.8375, 0.9467, 0.8444, and 0.8267 for NUS-WIDE database sizes of 200, 400, 600, and 800, respectively. It is observed from Figure 7 that the Proposed CGATSFRFOPSO method has a higher recall value than the existing CGATMDOPSO method.

**Figure 8:**
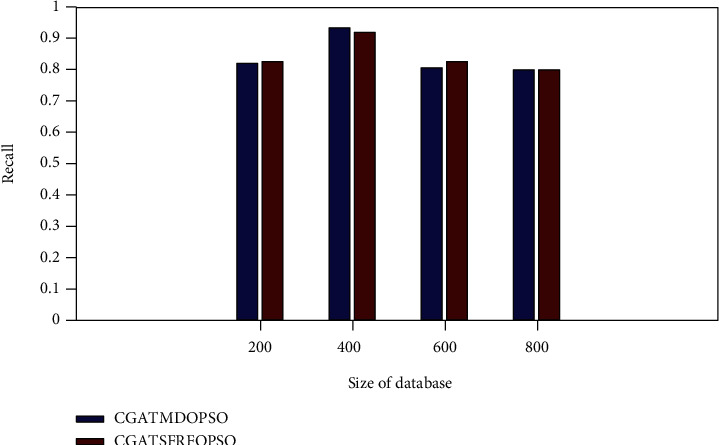
Recall rate comparison (PASCAL VOC).

Figure 8 exhibits the recall comparison of the proposed method with the CGATMDOPSO method. The proposed CGATSFRFOPSO approach gives the recall rate of 0.8262, 0.9200, 0.8253, and 0.8035 for PASCAL VOC image database sizes of 200, 400, 600, and 600, respectively. It is a superior recall rate to the existing method.

### 4.4. Execution time

**Figure 9:**
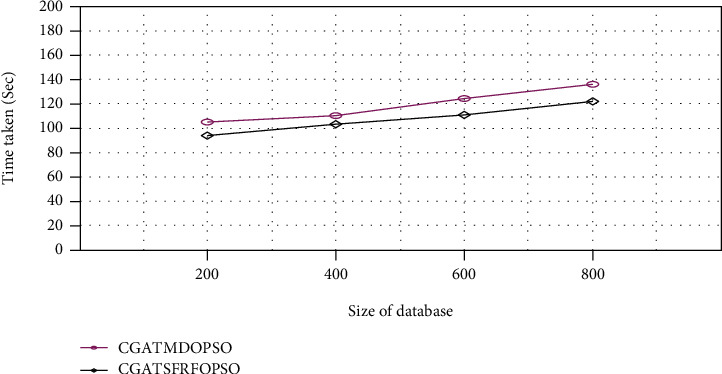
Execution time comparison (NUS-WIDE).

Figure 9 shows that the proposed CGATSFRFOPSO methodology provides a better result than the existing CGATMDOPSO approach with reduced execution time of 95, 105, 110, and 120 seconds for the NUS-WIDE image database sizes of 200, 400, 600, and 800, respectively.

**Figure 10:**
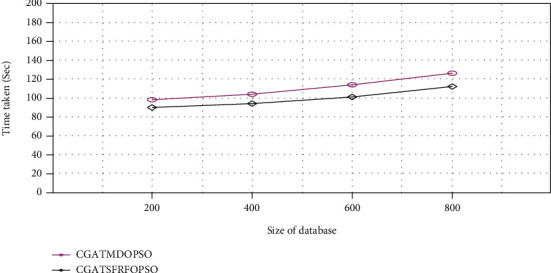
Execution time comparison (PASCAL VOC).

From Figure 10, it can be proved that the proposed methodology provides a better result than the existing approach by reducing execution time. The execution time of the proposed CGATSFRFOPSO method is decreased to 90, 95, 100, and 113 seconds for PASCAL VOC image database sizes of 200, 400, 600, and 800, respectively. From the experimental results, it be can be concluded that the proposed method is superior to the existing system.

### 4.5. Outputs of Proposed CGATSFRFOPSO Approach

**Figure 11:**
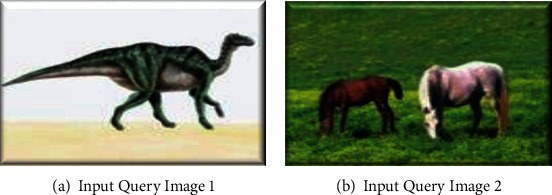
(a) Input query image 1 and (b) input query image 2.

**Figure 12:**
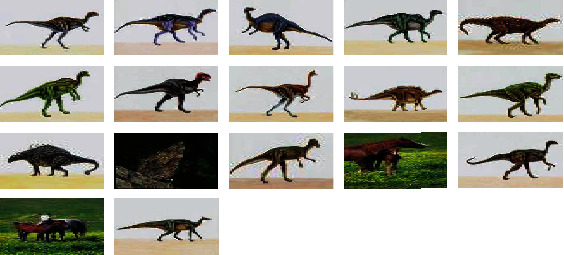
Output images for query image 1 using CGATSFRFOPSO.

**Figure 13:**
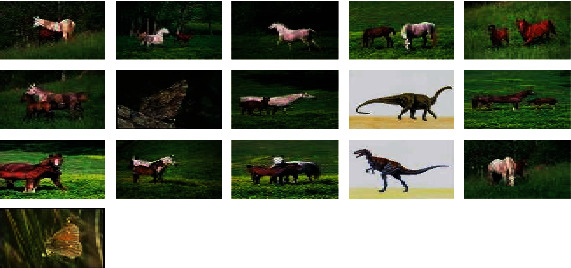
Output images for query image 2 using CGATSFRFOPSO.

The results of the proposed CGATSFRFOPSO approach for two input images are presented in this section.  Figure 11 shows the input images. Figures 12 and 13 show the retrieved output of image 1 and image 2, respectively. When compared to the results of the CGATMDOPSO approach, the resultant output of the proposed CGATSFRFOPSO is found to have better retrieval efficiency.

## 5. Conclusion

In this paper, the proposed CGATSFRFOPSO model is implemented for an efficient CBIR system by extracting additional shape features and shape descriptors. The contour-based shape feature extraction methods are used to extract the shape features and shape invariant features. These features have been selected and combined by using Particle Swarm Optimization (PSO) for better and more efficient retrieval of images from the image database. Finally, the input query image from the image database is retrieved based on the classification of training images by using the random forest classifier. As a whole, it can be concluded that the proposed CGATSFRFOPSO method provides efficient image retrieval results compared to the earlier work. The directions for future research of this research work are as follows:

- The proposed research work can be extended to multispectral images, hyperspectral images, super hyperspectral images, and remote sensing images
- Recent filtering techniques based on the Artificial Swarm Intelligence (ASI) may be used for feature extractions which may increase the overall performance of the system
- The techniques based on high-level semantic features like face recognition, biometric systems may be used to provide better-optimized results, which make use of genetic algorithms
- An effective soft computing technique like Neuro-Fuzzy techniques can be incorporated with the feature descriptor technique for better overall performance

## Figures and Tables

**Algorithm 1:**
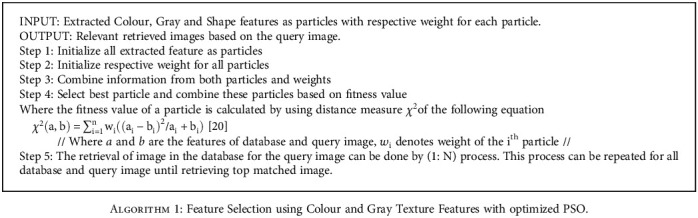
Feature Selection using Colour and Gray Texture Features with optimized PSO.

**Algorithm 2:**
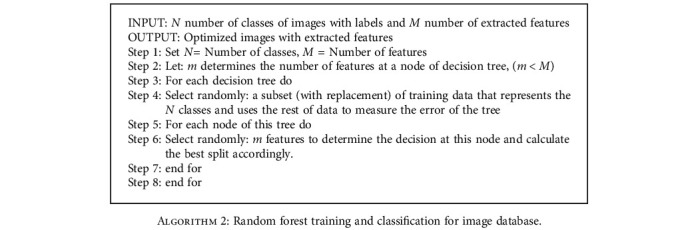
Random forest training and classification for image database.

**Table 1:** Performance Comparison of CGATMDOPSO and CGATSFRFOPSO (NUS-WIDE).

Performance metric NUS-WIDE Dataset	Size of database							
	CGATMDOPSO				CGATSFRFOPSO			
	200	400	600	800	200	400	600	800
Accuracy	82.5843	92.6667	83.6710	81.3084	83.7079	94.6667	84.4681	82.7103
Precision	0.8256	0.9267	0.8362	0.8128	0.8371	0.9479	0.8455	0.8263
Recall	0.8258	0.9267	0.8361	0.8141	0.8375	0.9467	0.8444	0.8267
Execution time	105	110	123	138	95	105	110	120

**Table 2:** Performance Comparison of CGATMDOPSO and CGATSFRFOPSO (PASCAL VOC).

Performance metric PASCAL dataset	Size of database							
	CGATMDOPSO				CGATSFRFOPSO			
	200	400	600	800	200	400	600	800
Accuracy	80.8989	92.6667	79.1489	78.0374	82.5843	92.0000	82.5532	80.3738
Precision	0.8092	0.9267	0.7915	0.7804	0.8258	0.9212	0.8258	0.8029
Recall	0.8095	0.9267	0.7915	0.7816	0.8262	0.9200	0.8253	0.8035
Execution time	98	105	115	125	90	95	100	113

## Data Availability

The datasets generated and/or analysed during the current study are available in the NUS-WIDE repository. Link: https://lms.comp.nus.edu.sg/wp-content/uploads/2019/research/nuswide/NUS-WIDE.html
